# 

**Published:** 1912-03-02

**Authors:** 


### The Commission on Sleeping-Sickness.

The Commission which the Government decided to send out for the study of sleeping-sickness to Nyasaland should achieve good results. Sir David Bruce is, of course, the leader, and with him, we may recall, are Major Harvey and Captain Hamerton, of the Royal Army Medical Corps, together with Mr. Newstead, of the Liverpool School of Tropical Medicine. The subject for decision is a very important one. Cases of sleeping-sickness have been occurring with increased frequency in Nyasaland and North-Eastern Rhodesia, and yet though carefully searched for, *Glossina palpalis*, the usual carrier of the infection, has not been discovered. It is possible that *Glossina morsitans*, another species of tsetse fly, may be responsible, and as this fly is extremely prevalent over large areas of the country the importance of deciding what part, if any, it plays in the dissemination of the disease is apparent.

Quite recently also an idea has become prevalent that the trypanosome found in these Rhodesian and Nyasaland cases is a different species from the parasite *Trypanosoma gambiense*, the causative agent of the disease in other parts of Central Africa, and this is a further question of the greatest importance. Whether this should ultimately turn out to be so or not there is no doubt about the toxicity of the Rhodesian strain of trypanosome, human cases and experimental animals dying with great rapidity. There is a wide field open for the Commissioners then in deciding these and other points, and the results of their work will be looked forward to with eagerness by workers in tropical medicine.

### PRACTICAL BOOKS FOR INSTITUTION WORKERS.

| | | s. | d. |
|---|---|---|---|
| Burdett's Hospitals and Charities. | net | 10 | 6 |
| The Cottage Hospital: Its Origin, History, Value and Advantages. | | 0 | 6 |
| Hospital Expenditure: The Commissariat. | | 2 | 6 |
| The Future of the Hospitals: A Scheme of Co-operation between the Patients, the Hospitals and the State. | | 0 | 2 |
| Sanatoria for the People. | net | 1 | 0 |

Ledgers, Account Books, &c., of all kinds for large or small Institutions.

THE SCIENTIFIC PRESS, LTD., 28-29 SOUTHAMPTON STREET STRAND, W.C.

### Florence Nightingale Memorial.

We have received the following contribution: Miss Clara Thomas (£10 for statue and £10 for annuities), £20.

The following is a list of contributions received by Mr. G. Q. Roberts since last week: Miss Emma Goldsmid, £10 10s.; Sir Edgar Speyer, Bart., £10 10s.; Mrs. Murray Smith, £10 10s.; I. F. W. Deacon, Esq., £10 10s.; collection amongst Mounted Infantry, Longmoor Camp, £10; 1st Leicestershire Regiment, £7; Royal Scottish Nursing Association, £5 5s.; James Horlick, Esq., J.P., D.L., £5 5s.; Ernest de la Rue, Esq., £5 5s.; J. Francis Mason, Esq., M.P., £5; Sir George Dalhousie Ramsay, £5; Lord Barrymore, £5; Miss E. Baird Johnstone, £5; Colonel R. Williams, M.P., £3 3s.; Lord Haversham, £3 3s.; 26th (Field) Company Royal Engineers, £3; staff of Louise Margaret Hospital, Aldershot, £2 2s. 6d.; Colonel H. S. E. Reeves, £2; Guild of St. Barnabas for Nurses (Bournemouth branch), £2; Mrs. E. Drake's collection, £1 17s.; Mrs. Harrison, £1 1s.; Miss A. M. Youngman, R.I., £1 1s.; J. S. Ainsworth, Esq., £1 1s.; Mrs. Neville, £1 1s.; nursing staff, Soho Hospital for Women, £1; Mr. and Mrs. Money, £1; R. H. Reade, Esq., J.P., D.L., £1; collected by Matron, Warneford Hospital, Leamington, £1; the Rev. Canon Rawnsley, £1; the Rev. Chr. Wordsworth, £1; Sister Mary Cameron (per Sir Henry Burdett) (annuities), 14s.; the Misses Baron (annuities), 3s.; Florence Norman, Florence Keable, and Florence Smalls, 3s.; Miss Clutton, 2s. 6d.; H. Curtler, Esq., 1s.; "A Reader of 'Filomena' in *Illustrated London News*," 1s.

### Appointments.

Mr. J. G. Hare has been appointed Demonstrator in Bacteriology, King's College, University of London.

The following appointments have been confirmed at the Manchester Royal Infirmary: Dr. A. E. Woodall, M.D., Ch.B., M.Sc. Vict., to be Medical Officer at the Central Branch; Mr. Kenneth Bean, M.B., Ch.B. Vict., and Mr. James Walker, M.B., Ch.B., Vict., to be House Physicians; Mr. E. Grey, M.B., Ch.B. Vict., and Mr. E. Moritz, M.B., Ch. B. Vict., to be Senior House Surgeons; and Mr. G. E. Sawdon, M.B., Ch.B. Vict., to be Junior House Surgeon. Dr. C. E. Lea, M.D., has been reappointed Assistant Director of the Clinical Laboratory.

### Gifts and Bequests.

Mrs. Sarah Williamson, of Blackpool, has bequeathed £500 to the Victoria Hospital, Blackpool, and £200 each to the following institutions in Manchester : The St. Mary's Hospital, the Lying-in Hospital, Deansgate, the Royal Eye Hospital, and £200 to the Northern Counties Hospital and Home for Incurables at Heaton Mersey.

Under the will of Mrs. Ellen Dyke Frost the Leicester Infirmary has received £1,000 for the General Fund and £1,000 for the Children's Hospital. And from the estate of the late Mr. Charles Turner, who died some four or five years since (from which estate a sum of £1,400 has already been received), a further sum of £933 odd (being two-thirds of the residue) has been paid by the executors to the Leicester Infirmary.

The London Homœopathic Hospital, Great Ormond Street, W.C., has received £1,000 from an anonymous lady to extend the Convalescent Home for men patients as well as for women and children as at present. The fund now reaches £1,600. About £3,000 will be required, and Colonel Clifton Brown, the Treasurer of the Home, has just sent a cheque for £500, and the Earl of Dysart has promised £100 if the remaining £800 is contributed so that the Home can be extended before the coming summer.

The Samaritan Free Hospital for Women has received £10 10s. from the Cutlers' Company.

Mr. Montague G. Jessett has left £100 to the Seamen's Hospital Society, and £100 to the Imperial Cancer Research Fund. The residue of the property, about £40,000, has been devoted to the King Edward's Hospital Fund.

Mr. Wm. Milward-Jones, of Co. Dublin, has bequeathed £1,000 to the Royal National Hospital for Consumption at Newcastle, Co. Wicklow. He also left £600 each to ten hospitals in Ireland, and the same amount to the Royal Victoria Eye and Ear Hospital.

The Royal National Hospital for Consumption, Ventnor, has received a subscription of twenty guineas from the King.

### The Week's Novelties.

### PRACTICAL TYPES OF INVALID FURNITURE.

One of the most attractive, indeed instructive, catalogues ever compiled is that recently issued by John Ward, Ltd., of Tottenham Court Road. In it the hospital secretary or the sanatorium superintendent can find every variety of practical invalid or resident staff furniture, from wedge-pillows to walking-machines. It is particularly to be noted that the invalid-chairs here described are not of that excessively complicated type often advertised. They are simple and sensible in aim. For any institutional official who wants to keep a file of all such appliances this new catalogue should be procured. Every type of invalid furniture can be studied practically in its pages.

### THE MINN-SLING (Reg. No. 589179).

We have received from the makers, Messrs. Maw, Son and Sons, a new and improved arm-sling, which has been invented and protected by Mrs. G. Vickerman Hewland, the wife of a practitioner in St. Leonards. The sling, which we have had the opportunity of putting to a practical test, appears to us to possess considerable advantages over the well-known triangular bandage for the purpose of supporting the arm, forearm, or hand. It is exceedingly light, being made of silk, and is sent out with a band having a simple twist which enables the sling to be supported most comfortably over the shoulder, while the buckle is so placed as not to cause any pressure on the neck. The Minn-sling may be used for either arm, and is easily adjusted to any required length. It affords good support to the elbow, is easily put on, and retains its position most effectively. We can cordially recommend the Minn-sling, which will be found a great boon to both ladies and gentlemen requiring a sling for any length of time. It may be had in colours to match a lady's gown, but is stocked by Messrs. Maw in black and navy-blue silk.

